# Striking lineage diversity of severe acute respiratory syndrome coronavirus 2 from non-human sources

**DOI:** 10.1016/j.onehlt.2021.100363

**Published:** 2021-12-16

**Authors:** Marina Muñoz, Luz Helena Patiño, Nathalia Ballesteros, Sergio Castañeda, Nicolás Luna, Lourdes Delgado, Carlos Hernandez-Pereira, Maryia V. Shaban, Shirly Alexandra Muñoz, Alberto Paniz-Mondolfi, Juan David Ramírez

**Affiliations:** a Centro de Investigaciones en Microbiología y Biotecnología-UR (CIMBIUR), Facultad de Ciencias Naturales, Universidad del Rosario, Bogotá, Colombia; b Instituto de Investigaciones Biomédicas IDB/Incubadora Venezolana de la Ciencia, Barquisimeto, Venezuela; c Centro de Tecnología en Salud (CETESA), Innovaseq SAS, Bogotá, Colombia; d Unidad de Salud de Ibagué (USI) E.S.E., Ibagué, Colombia; e Microbiology Division, Department of Pathology, Molecular and Cell-Based Medicine, Icahn School of Medicine at Mount Sinai, New York, USA

**Keywords:** SARS-CoV-2, Animals, Environment, Humans, Lineages, Alpha variant

## Abstract

Due to the necessity to control human-to-human spread of severe acute respiratory syndrome coronavirus 2 (SARS-CoV-2), the overwhelming majority of the generated data on this virus was solely related to the genomic characteristics of strains infecting humans; conversely, this work aimed to recover and analyze the diversity of viral genomes from non-human sources. From a set of 3595 publicly available SARS-CoV-2 genome sequences, 128 lineages were identified in non-human hosts, the majority represented by the variants of concern Delta (*n* = 1105, 30.7%) and Alpha (*n* = 466, 12.9%), followed by B.1.1.298 lineage (*n* = 458, 12.7%). Environment, *Neovison vison, Odocoileus virginianus* and *Felis catus* were the non-human sources with the highest number of lineages (14, 12 and 10, respectively). Phylogenomic analyses showed viral clusters from environmental sources, *N. vison*, *O. virginianus*, *Panthera tigris*, and *Panthera leo*. These clusters were collectively related to human viruses as well as all other non-human sources that were heterogeneously distributed in the phylogenetic tree. Further, the genetic details of viral genomes from bats and pangolins were independently investigated owing to their high divergence, revealing five distinct clusters. Cluster 4 exclusively included bat-sourced genomes and the SARS-CoV-2 reference strain Wuhan-01. In summary, this study identified new genetic landmarks of SARS-CoV-2 evolution. We propose potential interspecies transmission routes of SARS-CoV-2 between animals and humans, which should be considered in order to establish better pathogen surveillance and containment strategies.

## 1 Introduction

To date, research on severe acute respiratory syndrome coronavirus 2 (SARS-CoV-2) has been overwhelmingly focused on the study of viral interactions with human hosts. However, the study of isolates from non-human sources is equally useful, as this may provide vital insights into the process of interspecific transmission and the diversity of hosts and environmental ranges occupied by this virus. This would also shed light on the dynamics of cross-species transmission among high-density maintenance hosts, including potential crossing of viruses to new hosts (spillover) and reversion to their natural reservoirs (spillback) [[1], [2], [3], [4], [5], [6], [7], [8]].

A similar event of interspecific transmission, unique to SARS-CoV-2, defined the zoonotic/anthropozoonotic character of this virus [9]; therefore, such events are significant for epidemiological analysis, as they determine the nature, scale, and duration of the infection [10]. However, the relevance of such studies is not accompanied by easily accessible information. Indeed, viral genomic data from human isolates greatly outnumber zoonotically derived data. For instance, more than 5 million sequences of human-isolated viruses have been shared through the Global Initiative on Sharing All Influenza Data (GISAID) [11], the largest repository of SARS-CoV-2 sequences, while less than 4000 genomes from non-human sources have been deposited.

The global pandemic of the past year has had strong repercussions on healthcare and on the overall environmental, social, and economic systems. Therefore, the emergence of coronavirus disease (COVID-19) has reinforced the role of the scientific community in supporting the welfare of mankind. Conjointly, this community has answered questions pertinent to the pathophysiological mechanisms and genotypic signatures of SARS-CoV-2, and identified targets for the prevention and control of infection. Nevertheless, despite massive efforts, the rates of SARS-CoV-2 infection have remained a serious public health issue in many countries, and new lineages of the virus, such as the most recently designated Omicron variant of concern (VOC), continue to emerge as a consequence of its constant diversification. In this context, it is necessary to consistently investigate SARS-CoV-2 variants circulating in abiotic sources, and in animals different from humans, in order to identify early on possible propagation niches within novel trajectories of viral spread. Therefore, this study aimed to evaluate the epidemiological distribution, diversity, phylogenetic relationships, and mutation histories of SARS-CoV-2 lineages from zoonotic and abiotic niches. We attempted to establish a foundation for studying the emergence of SARS-CoV-2 lineages while effectively mapping likely ranges of future SARS-CoV-2 dispersal, to support measures countering future pandemic outbreaks before they arrest current progress.

## 2 Methods

### 2.1 Data retrieval

Non-human SARS-CoV-2 genome sequences were downloaded from the *Global Initiative on Sharing All Influenza Data* (GISAID) database [11]. Here, a quality control step was included to select exclusively for high-quality genomes for subsequent analysis, including low coverage and an increased number of Ns. A dataset with 3595 sequences was built including all entries until November 15, 2021. Compiled metadata for the analyzed dataset is included in Supplementary Table 1.

### 2.2 Quality control and typing of analyzed genomes

The set of genomes was typed using PANGOLIN (*Phylogenetic Assignment of Named Global Outbreak LINeages*) tool [12]. Lineages with less than five entries were grouped within the ‘other lineages’ category by major lineages (A and B). Those genomes that failed to type were included under the category ‘none’. The relative abundance for each lineage per non-human source was calculated considering the number of genomes per lineage compared to the total number of entries for each source. Additionally, genome diversity was estimated by non-human source, according to the number of lineages identified based on the total number of genomes reported for each.

### 2.3 Descriptive analyses

A descriptive analysis was conducted based on information included in the metadata for each of the downloaded genomes from the complete dataset (*n* = 3595 genomes). The most relevant categorical variables (lineage, geographic origin, and sample source) were analyzed using descriptive statistics, expressed in frequencies and proportions. Chi-square tests were applied in order to identify possible associations between variables of interest. Then, we estimated the degree of association using Cramér's V statistic software [13]. Multiple comparisons between different categories were made by implementing post hoc tests through the *chisq.posthoc.test* function included in the vcd package R software, which implements pairwise comparisons using Bonferroni as adjustment method. All statistical analyzes were carried out using the R software (RStudio Team 2019). All tests of significance were two-tailed, and *P*-values < 0.05 were considered statistically significant.

### 2.4 Phylogenomic analyses

Subsequently, we screened the established dataset (*n* = 3595 genomes) to analyze and infer phylogenetic relationships among all non-human sources. A comparative approach was ran including 2285 genomes from humans included as representative of SARS-CoV-2 lineages. These reference genomes were downloaded from Nextclade (https://clades.nextstrain.org/). The complete dataset used for phylogenomic analyses ascends to 5881.

### 2.5 Identification of mutation across genome sequence

A genome wide mutation detection analysis from non-human sources was completed. Here, Single Nucleotide Polymorphisms (SNPs), as well as Insertions and Deletions (InDels), were screened using a package within the Nextstrain tool (https://github.com/nextstrain/ncov). Identified mutations were graphically represented in a model to genome size scale, considering the coordinates in the genome of the Wuhan-01 (NC_045512) reference strain.

## 3 Results

### 3.1 Epidemiological profiles of SARS-CoV-2 genomes from non-human sources

**Fig. 1:**
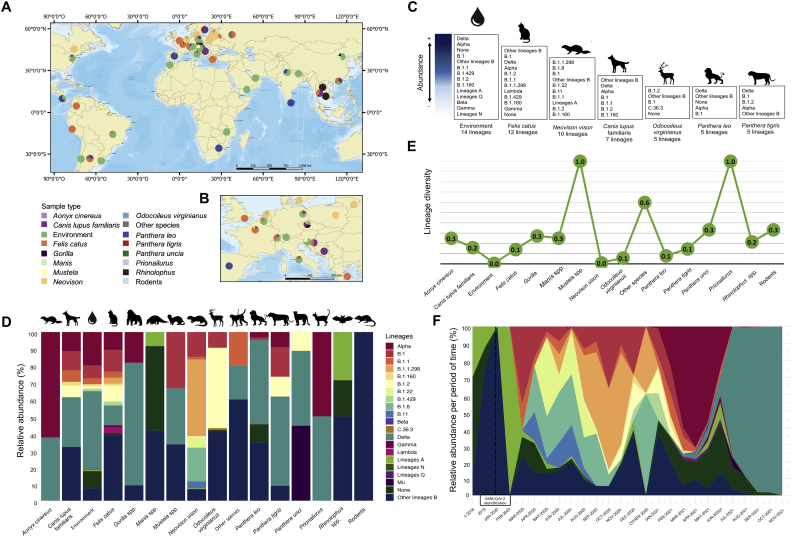
**Epidemiological profiles of SARS-CoV-2 genomes derived from non-human sources. A.** Map indicating the geographical origin of SARS-CoV-2 genomes from non-human sources reported worldwide. **B.** Magnification of the map depicting the origin of SARS-CoV-2 genomes in Europe, the region with the highest number of entries. **C.** List of SARS-CoV-2 lineages identified in each analyzed non-human source. The lineages were established using Phylogenetic Assignment of Named Global Outbreak LINeages (PANGOLIN) [12]. **D.** Relative abundance of SARS-CoV-2 lineages for each analyzed non-human source. Percentages were calculated considering the number of genomes assigned to each lineage with respect to the total number of genomes reported for each source. **E.** Lineage diversity for each analyzed non-human source. An index was calculated for each source considering the number of observed pangolin lineages with respect to the total number of reported genomes. **F.** Temporal variation of the relative abundance of the lineages, calculated considering the number of genomes reported for each lineage with respect to the total number of genomes per period of time. All epidemiological analyses were conducted using the complete dataset, including 1796 genomes.

By using metadata from the complete dataset (*n* = 3595), epidemiological analysis of zoonotically and abiotically sourced SARS-CoV-2 enabled to detect the virus in 16 non-human sources, the most frequent being the environment (*n* = 2217, 61.7%), followed by *Neovison vison* (*n* = 998, 27.8%), *Odocoileus virginianus* (*n* = 86, 2.4%), and *Felis catus* (*n* = 85, 2.4%). This analysis revealed a wide geographical distribution in this dataset, which included genomes from 47 countries, representing all continents except Oceania (Fig. 1A). The most of these genomes have been reported from European countries (Fig. 1B).

### 3.2 Heterogeneous profiles of SARS-CoV-2 lineages circulating in non-human sources

Qualitative analysis of the lineage amount recorded in each non-human source (Fig. 1C) revealed that environmental samples exhibited the highest number of lineages, with a total of 9 lineages plus other A, B, Q and N lineages, as well as a set of genomes that could not be assigned to any lineage and were hence included in the ‘none’ category. This abiotic source, widely distributed and with a high possibility of contact with humans, included multiple variants of concern (Delta, Alpha, Beta and Gamma). Detailed analysis of relative lineage abundance illustrated that the SARS-CoV-2 variants of this dataset were enriched in the variants of concern Delta (*n* = 1105, 30.7%) and Alpha (*n* = 466, 12.9%), followed by B.1.1.298 lineage (*n* = 458, 12.7%). As shown in Fig. 1D, the relative abundance of diverse lineages varied widely in wild and domesticated hosts, as well as in abiotic sources, without any observable pattern, although such variation was most similar to that of the B lineage. Notably, a high proportion of genomes detected in *Rhinolophus* spp. and *Manis* spp. were hence included in the ‘none’ category. A graphical analysis of lineage diversity in non-human sources (Fig. 1E) revealed that the highest diversity was observed in *Mustela* spp. and *Prionailurus*.

Finally, a temporal analysis (Fig. 1F) based on the relative seasonal abundance of dominant lineages showed changes in viral diversity during the time window of analysis, with initial predominance of the A and ‘none’ lineages among previously reported sequences for the identification of SARS-CoV-2 (from 2010 to 2019). The results of statistical tests validating the associations between SARS-CoV-2 lineages and non-human sources can be found in Supplementary File 2. Strikingly, during 2021 the proportion of variants of concern increased, initially from Alpha (from January to May) and more recently from Delta (from June to the date of this analysis).

### 3.3 Phylogenomic relationships of SARS-CoV-2 lineages from non-human sources

**Fig. 2:**
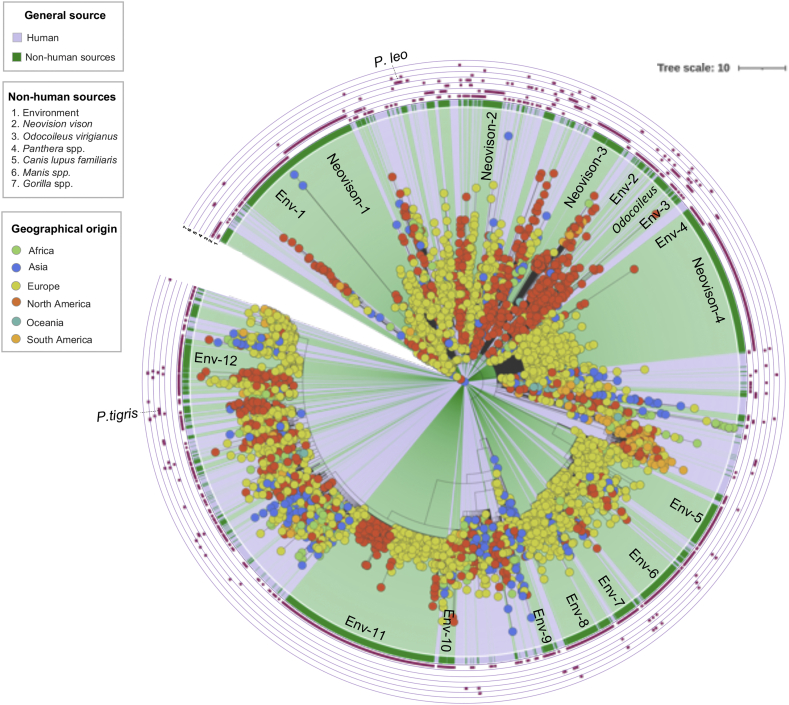
**Phylogenetic analysis showing the relationships among SARS-CoV-2 genomes from non-human sources, and between these genomes and those sourced from humans.** A total of 3656 sequences were included in this analysis, representing 1752 high-quality genomes derived from non-human sources (marked in green), 1902 reference genomes derived from humans (marked in gray), and the genome of the reference strain Wuhan-01 (NC_045512). The identity of the specific non-human sources is marked in the external rings, which are colored purple in correspondence of the respective non-human source listed in the legend. This analysis revealed that SARS-CoV-2 lineages circulating in non-human sources are heterogeneously distributed throughout the phylogenetic tree. Clusters were identified exclusively for the following five sources: the environment (nine clusters), *Neovison vison* (six clusters), *Mustela* spp., *Panthera tigris*, and *Panthera leo*, the latter three with one cluster each. SARS-CoV-2 genomes from the remaining sources were distributed randomly throughout the tree. Clusters from the same non-human source were related by geographical origin and subsequently closely related to reference genomes from humans. Six of the nine clusters identified for genomes sourced from the environment (Env-2 to Env-7) belonged to a well-differentiated cluster which also included human-sourced genomes; most of these corresponded to the Alpha variant (B.1.1.7). (For interpretation of the references to colour in this figure legend, the reader is referred to the web version of this article.)

A preliminary phylogenetic analysis revealed a set of 27 highly divergent genomes, whose relationships with the other genomes could not be resolved owing to insufficient branch resolution. These genomes, sourced from *Rhinolophus* spp. and *Manis* spp., were considered independent in subsequent analyses (Fig. 2).

The phylogenomic analysis with 5854. sequences (Fig. 2) included: a high-quality dataset of non-human origin, without the 27 sequences from *Rhinolophus* spp. and *Manis* spp., plus a reference dataset from humans (*n* = 1903), and the universal reference sequence Wuhan-01 (NC_045512).

This disclosed that SARS-CoV-2 circulating in the non-human source is heterogeneously distributed throughout the tree. Clusters were identified exclusively for the following five sources: Environment (12 clusters), *N. vison* (4 clusters), *Odocoileus virigianus*, *P. tigris*, and *P. leo*—the latter three with one cluster each. The remaining sources were distributed indistinctly throughout the tree. The clusters from the same non-human source were related by geographical origin and subsequently closely related to reference genomes from humans. Five of the 12 clusters identified for the ‘Environment’ category (Env-5 to Env-8), appertained to a well-differentiated cluster in which they were found related to human genomes (most corresponding to the variant alpha B.1.1.7) (Fig. 2, right).

### 3.4 Genomic variations across SARS-CoV-2 lineages from non-human sources

**Fig. 3:**
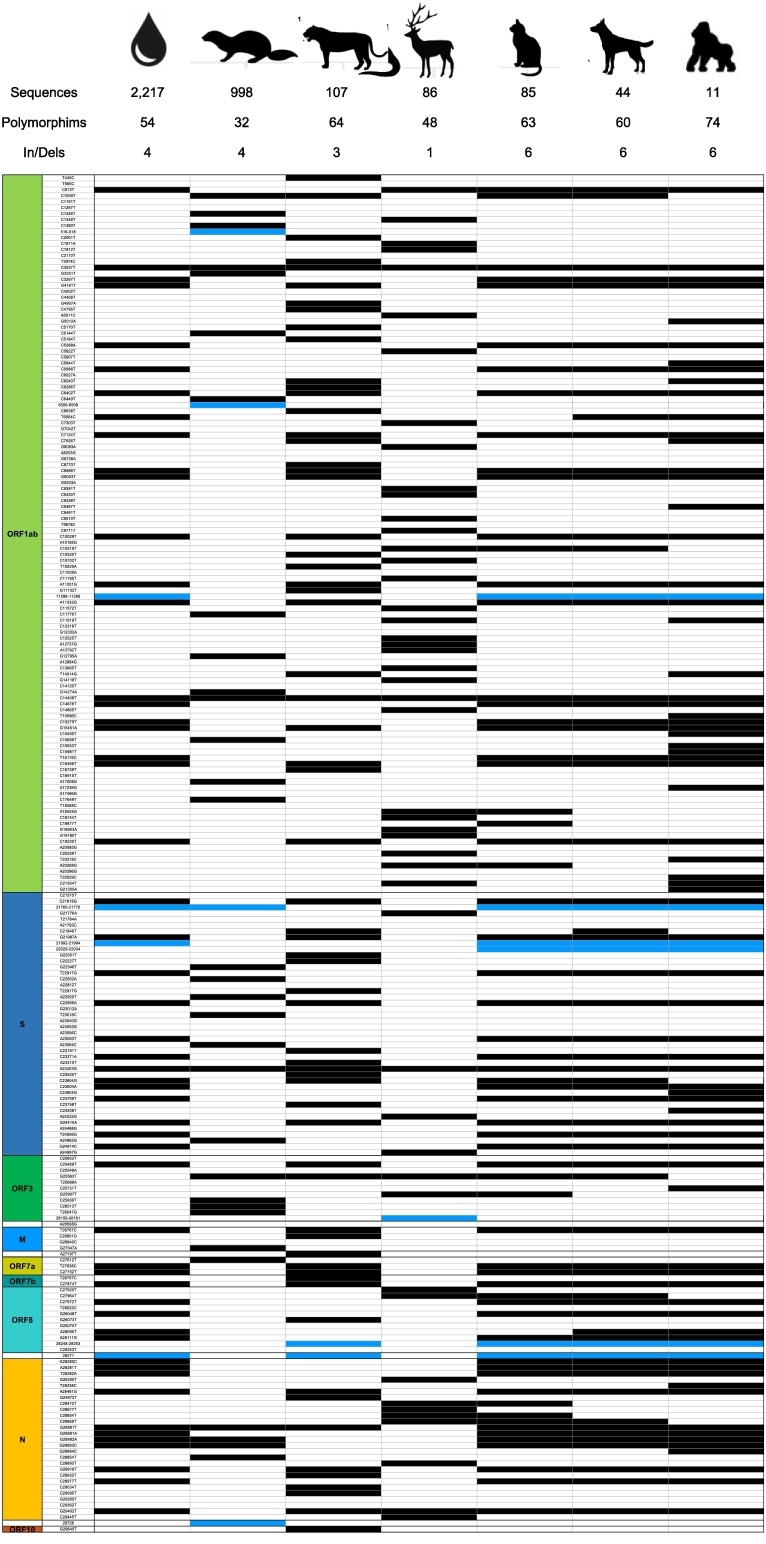
**Whole-genome mutation profiles of SARS-CoV-2 lineages from non-human sources.** The mutations were represented graphically considering the genomic coordinates of the SARS-CoV-2 reference strain Wuhan-01 (NC_045512). Black lines represent the polymorphic sites while the blue lines represent Insertion/Deletions (In/Dels) across whole genome. (For interpretation of the references to colour in this figure legend, the reader is referred to the web version of this article.)

The analysis of mutations across the whole genome of each non-human source with more of ten genomes, compared with that of the reference strain Wuhan-01, revealed the presence of 32 to 74 polymorphic sites in each non-human-sourced genomes (Fig. 3**,** black lines). The non-human source displaying the highest number of polymorphic sites was *Gorilla* with 74, despite of the low number of samples (*n* = 11). *Panthera* spp. ranked second, with 64 polymorphic sites and 107 sequences available. The non-human source with the lowest number of polymorphic sites was *N. vison* with 32. Interestingly, genomes from all non-human sources exhibited three mutations frequently reported also in human-sourced viruses (red lines), namely C3037T, C14408T, and A23403G. The latter corresponds to D614G. Other SNPs were distributed across the genome, predominantly in the *S* and *N* open reading frames (ORFs). Conversely, insertions and deletions (InDels) were detected (Fig. 3**,** blue lines), revealing that the highest number was six and it was present in *F. catus*, *Canus lupus familiaris* and *Gorilla* spp., showing common profiles predominantly in the *S* ORF.

### 3.5 Phylogenetic relationships of SARS-CoV-2 lineages circulating in bats and pangolins

**Fig. 4:**
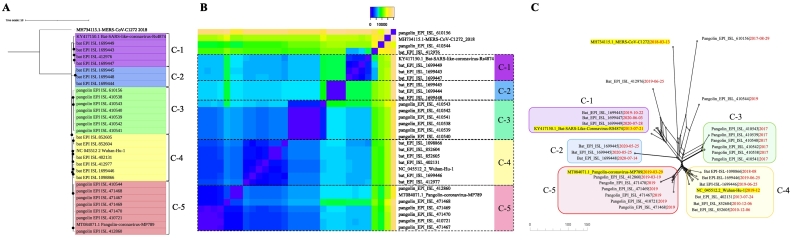
**In-depth analysis of clusters sourced from bats and pangolins.** Twenty-seven SARS-CoV-2 genome sequences from these two non-human sources were independently analyzed because of their high divergence from the other genomes considered. The genome sequences of the following strains were included as references: SARS-CoV-2 strain Wuhan-01 (NC_045512), bat SARS-like coronavirus strain Rs4874 (KY417150.1), pangolin coronavirus strain MP789 (MT084071.1), and MERS-CoV coronavirus strain C1272/2018 (MH734115.1). **A.** Phylogenetic analysis based on whole-genome alignment using Nextstrain [11]. **B.** Pairwise comparison of SNP distances after whole-genome alignment among the evaluated genomes. Clusters were marked on a SNP heatmap obtained in heatmapper (http://www.heatmapper.ca/). **C.** Phylogenetic network based on whole-genome alignment constructed in SplitsTree v5 using the neighbor-net algorithm [16]. We conducted an in-depth analysis of sequences from bats and pangolins in order to clarify the phylogenetic relationships among SARS-CoV-2 genomes from these two non-human sources (Fig. 4). This analysis included the 27 sequences excluded from global analysis, as they were highly divergent from the other sequences analyzed (Supplementary Fig. 1). The genome sequences of the following strains were included as references: SARS-CoV-2 strain Wuhan-01 (NC_045512), bat SARS-like coronavirus strain Rs4874 (KY417150.1), pangolin coronavirus strain MP789 (MT084071.1), and MERS-CoV coronavirus strain C1272/2018 (MH734115.1), as described in the Methods section. **In-depth analysis of clusters sourced from bats and pangolins.** Twenty-seven SARS-CoV-2 genome sequences from these two non-human sources were independently analyzed because of their high divergence from the other genomes considered. The genome sequences of the following strains were included as references: SARS-CoV-2 strain Wuhan-01 (NC_045512), bat SARS-like coronavirus strain Rs4874 (KY417150.1), pangolin coronavirus strain MP789 (MT084071.1), and MERS-CoV coronavirus strain C1272/2018 (MH734115.1). **A.** Phylogenetic analysis based on whole-genome alignment using Nextstrain [11]. **B.** Pairwise comparison of SNP distances after whole-genome alignment among the evaluated genomes. Clusters were marked on a SNP heatmap obtained in heatmapper (http://www.heatmapper.ca/). **C.** Phylogenetic network based on whole-genome alignment constructed in SplitsTree v5 using the neighbor-net algorithm [16]. We conducted an in-depth analysis of sequences from bats and pangolins in order to clarify the phylogenetic relationships among SARS-CoV-2 genomes from these two non-human sources (Fig. 4). This analysis included the 27 sequences excluded from global analysis, as they were highly divergent from the other sequences analyzed (Supplementary Fig. 1). The genome sequences of the following strains were included as references: SARS-CoV-2 strain Wuhan-01 (NC_045512), bat SARS-like coronavirus strain Rs4874 (KY417150.1), pangolin coronavirus strain MP789 (MT084071.1), and MERS-CoV coronavirus strain C1272/2018 (MH734115.1), as described in the Methods section.

Five main clusters, named C1 to C5, were detected in the phylogenetic tree (Fig. 4A); of these, three and two represented SARS-CoV-2 lineages from bats (C1, C2, and C5) and pangolins (C3 and C4), respectively. Clusters C4 and C5 were the most closely related. Interestingly, the reference sequence for SARS-CoV-2 (Wuhan-01) was included in cluster C4, a bat-related clade including SARS-CoV-2 sequences reported in 2010. Cluster C5 included exclusively SARS-CoV-2 sequences from pangolins together with their corresponding reference sequence (pangolin MP789).

The cluster more closely associated to the aforementioned clusters C4 and C5 was C3, including only sequences from pangolin. The remaining two clusters (C1 and C2), which appeared to be ancestral to clusters C3–C5, exclusively included bat-derived sequences. The existence of these clusters was confirmed: i) by calculating pairwise SNP distances from whole-genome alignments (excluding untranslated regions), in which fewer than 250 SNPs were observed among members of the same cluster (Fig. 4B); and ii) by reconstructing phylogenetic networks, in which the five clusters were also identified and resulted similarly divergent (Fig. 4C).

Such analysis revealed the occurrence of reticulation events, manifested in recombination signatures among the analyzed clusters. In both analyses, we identified three samples (two from pangolins and one from bat) that were excluded from any cluster, similar to the Middle East respiratory syndrome coronavirus (MERS-CoV) genome, which was included as an outgroup. For these genomes, the mutation profiles analyses were not conducted using the same scheme as for other non-human-sourced lineages. Each cluster exhibited more than 1000 mutations and multiples InDels with respect to the SARS-CoV-2 reference strain Wuhan-01. These were not evenly distributed across the whole genome; on the contrary, we remarked peculiar rearrangements in some regions. For example, a deletion in the *S* ORF of cluster C1 could be attributed to its closest reference (Supplementary Fig. 2). Moreover, the inclusion of sequences exclusively derived from bats in the C4 cluster, together with that of the reference SARS-CoV-2 strain Wuhan-01, revealed interesting information on the evolutionary history of this virus.

## 4 Discussion

The analysis of SARS-CoV-2 lineages distribution using genomic surveillance has revealed that the map of viral diversification does not trace, but rather overlaps with that of modern political boundaries and commercial routes. Yet, monitoring these overlays is crucial to predicting future dynamics of disease spread in the context of the COVID-19 pandemic. The analysis of pangolin lineages [12] has emerged as one of the most efficient strategies to follow viral diversification because of their high phylogenetic resolution. It is also possible to identify lineages capable of rapid expansion. Conversely, some lineages exhibiting clonal replication patterns can be confined to delimited areas, as demonstrated by Worobey et al., who studied the spread of SARS-CoV-2 in Europe and North America [17]. Previous studies identified viral variants of epidemiological concern (VOCs), such as the Alpha and Delta variants (B.1.1.7 and B.1.617.2 lineages, respectively), almost as quickly as they emerged in the population [18]. Similarly, this study examined the zoonotic and abiotic lineages that provided the blueprint for the current pandemic. We propose that genomic surveillance can be used as a tool for monitoring the dispersal routes of these new variants, and to promptly respond to the emergence of new VOCs, which would exacerbate the current pandemic.

Little is known about SARS-CoV-2 lineages circulating in wild and domesticated hosts, as well as in abiotic sources [19]. Nevertheless, our study revealed the circulation of SARS-CoV-2 in different wildlife species (Fig. 1), such as the American mink, which may transmit the largest diversity of lineages. Most of the samples revealing such outstanding genomic diversity of SARS-CoV-2 in mink (*N. vison*) can be traced to three Danish farms, where close contact with humans facilitates viral access to a series of susceptible mammalian hosts [20]. Other wild animals, including *P. tigris*, *Panthera leo*, and a range of primates, were found to be prone to infection by more than one SARS-CoV-2 lineage. In addition, viral strains were amply propagated in the domestic animals *F. catus* and *Canis lupus familiaris* (Fig. 1D). More recently, rodents as well as other animals offer a plausible theory to the emergence of the latest variant of concern, Omicron, which could have evolved in an animal reservoir before spilling back to humans [21].

Phylogenetic reconstruction (Fig. 2) and analysis of mutation profiles (Fig. 3) revealed that several clusters associated with sylvatic transmission were related to genomes previously isolated from humans. These findings align with those of the few available reports on wild and domesticated animals, which have proposed a parallel course of infection and disease in human- and animal-based viral cycles [21]. Together with these reports, our study provides the first line of evidence for the co-circulation of SARS-CoV-2 in wild animals and humans in the same venues, suggesting an animal-to-human transmission scenario, which can rapidly contribute to the phenotypic differentiation and enlarged pathological spectrum of the virus. Consistently, current clinical and research advisory boards advocate for the isolation of humans from house pets and other animals during the course of SARS-CoV-2 infection [22]. It would also be beneficial to examine the temporal dynamics and ecological aspects of SARS-CoV and MERS-CoV, since these seem to be similar to those related to the ever-progressive evolutionary history of SARS-CoV-2.

Today, we know that SARS-CoV and MERS-CoV likely emerged from bats as primary hosts [23,24], and then underwent interspecific transmission events to other animals as intermediate hosts and finally to humans. Palm civets became the intermediate hosts for SARS-CoV, while dromedary camels became the main intermediate reservoir for MERS-CoV [23]. It is likely that other mammals have served as intermediate amplifying hosts for these viruses, further favoring interspecies exchange while driving adaptation to humans. However, in our current study, the limited sampling of animal reservoirs compared to that of human sources was an important limitation. Therefore, further wide-ranging sampling of SARS-CoV-2 in various animal species is crucial for a better definition of the evolutionary pathways of SARS-CoV-2 and a better understanding of the ecological drivers and implications of host switching events across the human-animal interface.

Abiotic factors are also likely to play a role in the process of disease transmission, especially during the COVID-19 pandemic [25]. For instance, viral sequences with a truly remarkable lineage diversity were identified in the Austrian sewage system, consistent with other examples of viral persistence in a wide range of environmental matrices and surfaces [26]. In this environmental source, the most extensive lineage diversity was associated with the B.1.1, B.1.1.7, B.1.160, and B.1.258 lineages. The detection of the B.1.1.7 lineage, also known as the Alpha variant, is of substantial interest, as this is one of the most remarkable VOCs due to its increased transmissibility, virulence potential, and range of dispersal around the world [27,28]. Moreover, the lineages L3 and N.4 were exclusively found in this source.

Therefore, it is safe to say that SARS-CoV-2 has overcome the barriers that arrest the success of most pathogens in nature. Its emergence within the human-animal-environment circuit represented an opportunity to simultaneously reach a massive number of hosts, as blank canvases for developing novel mutations and phenotypic signatures defining ever-newer lineages of the original strain [29]. Host switching nurtures mutations that improve fitness, such as those on the Spike protein, which are most frequent and critical [30]. In this study, we identified genomes that could not be assigned to any established SARS-CoV-2 lineage, according to Phylogenetic Assignment of Named Global Outbreak LINeages (PANGOLIN). These were thus assigned to the ‘none’ category (Fig. 1D). Such lineages were found only in *Manis* spp. (pangolins) and *Rhinolophus* spp. (bats). Moreover, a high divergence was observed among them in a preliminary phylogenetic analysis (Supplementary Fig. 1). Therefore, these lineages were analyzed independently (Fig. 4).

Five clusters revealed genomic structural complexity. However, only the cluster C4 was found to be closely related to the established SARS-CoV-2 reference genome, Wuhan-01; this clustered with SARS-CoV-2 genomes from bats, some of which were deposited before the onset of the pandemic (since 2010; Fig. 4C). Such evidence demonstrates that bat SARS-CoV-2 lineages are very close phylogenetically to those derived from humans, so much that they were assigned to the same cluster. This finding may be explained in two possible ways: on the one hand, the virus may have conserved high transmission capacity between animals (in this case, bats); on the other hand, it could have undergone reverse zoonotic transmission, that is, the act of regularly crossing the host species boundary [31]. This was the case with the H1N1 influenza A virus, triggering the 2011 pandemic [32]. Such ‘spillback’ events represent a strategy for enhanced spatial dispersal [8]. A third hypothesis explaining these findings would be that SARS-CoV-2 may have been transmitted from bats to humans through a spillover event. To determine which of these hypotheses is the most accurate, it is necessary to analyze a larger number of SARS-CoV-2-like viral genomes from bats, pangolins, and a variety of morphologically similar hosts.

The composition of the remaining clusters (C1–C4) is consistent with the most robust hypothesis regarding the evolution of SARS-CoV-2 [30], which states that bat-derived lineages represented the primary ancestors (clusters C1 and C2) and pangolins acted as intermediate hosts (here, clusters C3 and C4, the most closely related to C5). We also detected critical mutations, such as a deletion in the *S* ORF in cluster C1 (Supplementary Fig. 1). However, considering that the genomes of the SARS-CoV-2 lineages isolated from pangolins clustered apart from those of bat-derived lineages and the reference genome Wuhan-01, we suggest that pangolin did not play a fundamental role as an intermediary host for viral maintenance, but rather that a direct spillover event from bats to humans occurred somewhere along the evolutionary history of these lineages. It could also be deduced that pangolins were only accidental hosts of SARS-CoV-2 and were not relevant for human transmission, as previously thought. This hypothesis is supported by the lack of convergent evolution of pangolin-derived SARS-CoV-2 genomes, which is instead well recognized in bat- and human-derived genomes [33]. Future studies are required to unveil the true role of pangolins and bats in the epidemic transmission of SARS-CoV-2 to humans.

In conclusion, this work sheds light on the intricate diversity of SARS-CoV-2 lineages circulating in the zoonotic and abiotic domains. We present here the first study related to the infection potential of this emerging and evolving pathogen. During the continued global COVID-19 pandemic, it will be fundamental to investigate this undocumented zoonotic SARS-CoV-2 pool, which, out of necessity, has hitherto been neglected. To this purpose, more non-human-sourced SARS-CoV-2 genomes need to be sequenced to pinpoint potential spillover events resulting in the emergence of novel antrhropozoonotic infectious agents. Such information would also contribute to the development of COVID-19 vaccines and the characterization of mutants that are likely to arise as the virus continues to travel from humans to animals to the environment, and vice versa [34].

The following are the supplementary data related to this article.

Supplementary Fig. 1 Supplementary Fig. 1

Supplementary Fig. 2 Supplementary Fig. 2

Supplementary File 1 Supplementary File 1

## Funding

This project was funded by the Universidad del Rosario in the framework of its strategic plan RUTA2025. Thanks to President and the University council for leading the strategic projects. We thank all investigators around the world that have deposited their genome sequences on GISAID. We also thank the Colombian network of SARS-CoV-2 genomic surveillance led by the National Institute of Health.

## Contributions

M.M. and J.D.R. conceived the study. M.M. and N.B. download the data. N.B. determined the relative abundances of lineages. S.C. developed the statistical analyses. N.L. analyzed the geographical distribution of sources and lineages. L.H.P. did the analysis of mutations across the genome. L.D., C·H and M.V.S. gave advice in lineage diversity analysis. M.M. and S.A.M. analyzed the data and results. M.M. and J.D.R drafted the manuscript. A.P.M. and J.D.R. critically revised the manuscript. All authors read and approved the final manuscript.

## Declaration of Competing Interest

The authors declare no competing interests.
