# Supplementary figures and images for: Striking lineage diversity of severe acute respiratory syndrome coronavirus 2 from non-human sources
Source: One Health. 2021 Dec 16;14:100363. doi: 10.1016/j.onehlt.2021.100363 (PMC8673956; doi:10.1016/j.onehlt.2021.100363)

Region ^

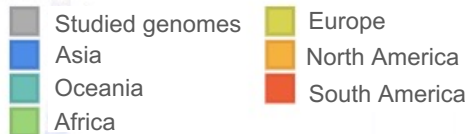

Divergent genomes

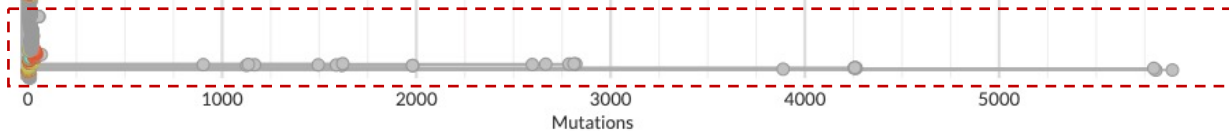

Supplement: Supplementary Fig. 1 [file mmc1.pdf]
