# Supplementary material for: Striking lineage diversity of severe acute respiratory syndrome coronavirus 2 from non-human sources
Source: One Health. 2021 Dec 16;14:100363. doi: 10.1016/j.onehlt.2021.100363 (PMC8673956; doi:10.1016/j.onehlt.2021.100363)
Supplement: Supplementary Fig. 2 [file mmc2.pdf]

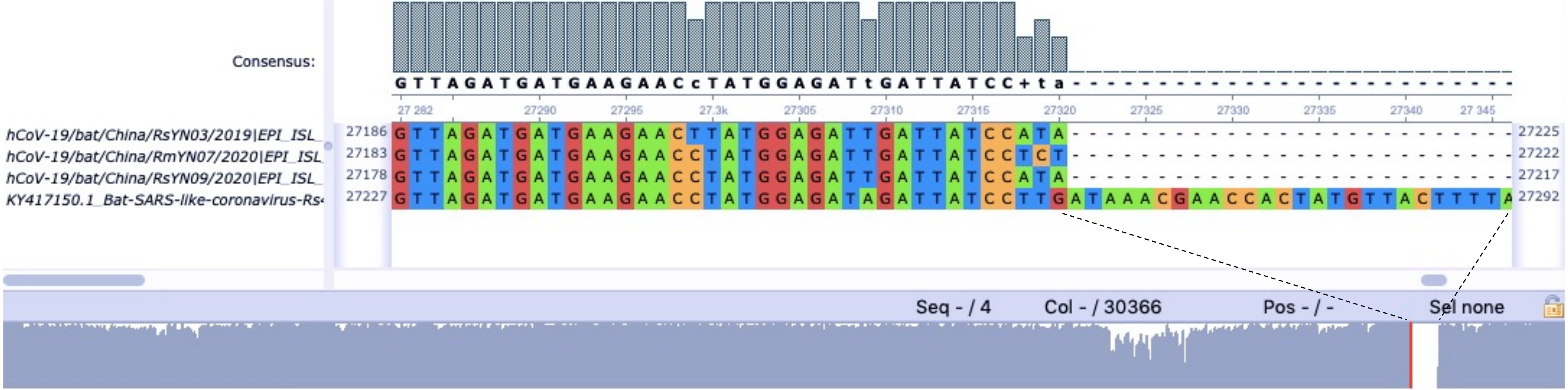
Consensus:
GTTAGATGATGAAGAACcTATGGAGATtGATTATCC+ta
hCoV-19/bat/China/RsYN03/2019|EPI_ISL_
hCoV-19/bat/China/RmYN07/2020|EPI_ISL_
hCoV-19/bat/China/RsYN09/2020|EPI_ISL_
KY417150.1_Bat-SARS-like-coronavirus-Rs
27186
27183
27178
27227
27225
27222
27217
27292
Seq - / 4
Col - / 30366
Pos - / -
Sel none
